# MYB transcription factor family in sweet cherry (*Prunus avium* L.): genome-wide investigation, evolution, structure, characterization and expression patterns

**DOI:** 10.1186/s12870-021-03374-y

**Published:** 2022-01-03

**Authors:** Irfan Ali Sabir, Muhammad Aamir Manzoor, Iftikhar Hussain Shah, Xunju Liu, Muhmmad Salman Zahid, Songtao Jiu, Jiyuan Wang, Muhammad Abdullah, Caixi Zhang

**Affiliations:** 1 Department of Plant Science, School of Agriculture and Biology, Shanghai Jiao Tong University, Shanghai, China; 2 School of Life Sciences, Anhui Agricultural University, Hefei, 230036 China

**Keywords:** MYB transcription factors, *PavMYB* genes, Phylogenetic analysis, Expression pattern, Dormancy

## Abstract

**Back ground:**

MYB Transcription factors (TFs) are most imperative and largest gene family in plants, which participate in development, metabolism, defense, differentiation and stress response. The MYB TFs has been studied in various plant species. However, comprehensive studies of MYB gene family in the sweet cherry (*Prunus avium L.*) are still unknown.

**Results:**

In the current study, a total of 69 MYB genes were investigated from sweet cherry genome and classified into 28 subfamilies (C1-C28 based on phylogenetic and structural analysis). Microcollinearity analysis revealed that dispersed duplication (DSD) events might play an important role in the MYB genes family expansion. Chromosomal localization, the synonymous (Ks) and nonsynonymous (Ka) analysis, molecular characteristics (pI, weight and length of amino acids) and subcellular localization were accomplished using several bioinformatics tools. Furthermore, the members of distinct subfamilies have diverse *cis*-acting regions, conserved motifs, and intron-exon architectures, indicating functional heterogeneity in the MYB family. Moreover, the transcriptomic data exposed that MYB genes might play vital role in bud dormancy. The quantitative real-time qRT-PCR was carried out and the expression pattern indicated that MYB genes significantly expressed in floral bud as compared to flower and fruit.

**Conclusion:**

Our comprehensive findings provide supportive insights into the evolutions, expansion complexity and functionality of *PavMYB* genes. These *PavMYB* genes should be further investigated as they seem to be brilliant candidates for dormancy manipulation in sweet cherry.

**Supplementary Information:**

The online version contains supplementary material available at 10.1186/s12870-021-03374-y.

## Background

The transcription factors (TFs) (sequence-specific DNA-bindings factor) are the proteins which regulates the rate of transcription of genetic information from DNA to messenger RNA through linkage with a specific DNA sequence. TFs control genes by turning them on and off to ensure that they are expressed in the right cell at the correct time and in the specific amount during the cell’s and organism’s lives [1, 2].Transcription factors (TFs) are crucial regulators of gene transcription along with at a DNA-binding domain, nuclear localization signal transactivation domain and oligomerization site.

One of the largest TFs families in the plant kingdom has been classified as the MYB gene family [3]. MYB proteins characterize the major transcription factor families in the plant kingdom. V-MYB was identified as first MYB TF in the avian myeloblastosis virus [4]. c-MYB-like TF is was the first plant MYB TF which was discovered in *Zea mays* and also justified for anthocyanin biosynthesis, [5]. Three types MYBTFs (A-MYB, B-MYB and c-MYB) were later discovered in many slime molds, fungi, insects and vertebrates [6].

MYB proteins are characterized by the presence of a highly conserved MYB DNA-binding domain at the N-terminus. The MYB domains normally consists of up to four imperfect amino acid sequence repeats (R) of about 50–53 amino acids and forming three alpha–helices. The 2nd and 3rd helices of individual repeat build a helix–turn–helix (HTH) structure with three frequently spaced tryptophan residues, which creates a hydrophobic core [7]. The 3rd helix of each repeat was recognized as the DNA recognition helix that approach directly to DNA [8]. Two MYB repeats are densely compacted in the central groove during DNA interaction and allowing the two recognition helices to attach to the unique DNA recognition sequence motif cooperatively. MYBs are classified into four categories based on the presence of 1–4 MYB repeats in sequence: 1RMYB-, R2R3MYB, 3RMYB-, and 4RMYB [9–11]. Gene family identification is now possible at genomic level due to presence of gene sequences. The previous studies indicate that more than 80 R2R3MYB TFs genes are identified in maize genome [12, 13]. The *Oryza sativa* genome contains following number of genes in each category such as, 1R-MYB (62 genes), R2R3-MYB (88 genes) and 3R-MYB (4 genes) [14], while in *Arabidopsis thaliana*,1R-MYBs (52 genes), R2R3-MYBs (135 genes) and 3R-MYBs (5 genes) were found [12]. MYB proteins are involved in secondary metabolism [15], hormone signal transduction, and response to environmental stress [16], cell differentiation and cell cycle [17]. The functions of MYB proteins are comprehensively studies in wide range of plants such as, *Malus domestica* [18], *Fragaria vesca* [19], *Arabidopsis thaliana* [20], *Pyrus bretschneideri* [21], *Gossypium raimondii* [22], *Solanum tuberosum* [23], *Solanum lycopersicum* [24] and *Actinidia chinensis* [25]. In *Dendrobium hybrida*, *DhMYB1* played vital role in the control of floral epidermal cell shape [26]. *SmMYB44* gene may improve bacterial wilt resistance in the eggplant [27]. *MdMYB124* and *MdMYB88* control the cold hardiness, and also improve drought tolerance by controlling cell walls and root vessels in apple [28, 29].

Dormancy (temporary suspension of growth) is a controlled process that regulates plant growth/ and development [30]. Paradormancy, endodormancy, and ecodormancy are three different kinds of dormancy processes. Paradormancy occurs when signals from other plant organs prevent meristem growth (for example, in buds or seeds). The shoot apex, for example, may prevent axillary buds from growing out by exerting apical dominance; but, when the apex is removed, this condition of paradormancy is disrupted, and axillary buds grow out. Endodormancy is a phase in which signals inside the meristem suppress meristem growth. In plants, vegetative buds usually become endodormant in the fall and early winter to overcome the cold stress, and extended phases of chilling (i.e., temperatures slightly above freezing) are needed before growth can resume, even under favorable environmental conditions. Plants remained in ecodormant even after endodormancy has been released due to severe environmental conditions such as cold or drought, which enable cell division and cell elongation [30]. Environmental stress responsive (Short days (SD), cold) and hormonal responsive (ethylene, gibberellin (GA), and abscisic acid (ABA)) are the indicators that have a direct role in growth limitation and bud formation [31, 32]. SD and low night temperatures in the autumn trigger growth suspension and promote vegetative bud set or shoot-tip abscission which is critical initial step of cold adaptation in many trees and other perennial plants [33, 34]. Cold temperatures may halt growth and induce endodormancy in certain species such as pear and apple [31, 35]. Previous studies proved that MYB genes are involved in dormancy regulation like *comp100540_c0_seq1, comp76266_c0_seq2, comp62057_c0_seq1* expressed their peak expression in ecodormancy and endodormancy in *Camellia sinensis* [36]. Same phenomenon was also confirmed in *Populus*. During short-day induction or dormancy transition, MYB62 and MYB4 exhibited distinct expression patterns [33, 37] while MYB96 is involved in seed dormancy regulation in Arabidopsis [38].

Dormancy is extensively influenced by external temperature, while bud break and flowering time are seriously affected by global warming. In the northern hemisphere, bud break and blooming dates are delayed in apple, cherry, birch and oak [39], while inadequate cold accumulation all through winter may result to inadequate dormancy release. These phenological variations have a direct effect on fruit crop production, potentially resulting in significant economic losses [40]. As a result, in order to combat fruit losses and anticipate future production changes, a better knowledge of bud responses to temperature stimuli in the context of climate change becomes critical. In recent years, numerous studies have used RNA sequencing technologies to evaluate the physiological and molecular processes of bud dormancy transitions in perennials. Sweet cherry (*Prunus avium* L.) is a perennial plant that is very temperature sensitive [35].

To explore the MYB effect on dormancy in sweet cherry, a genome-wide analysis of the MYB family in sweet cherry was carried out in this study, which included database searches using the *PavMYB* gene model and phylogenetic relationships, gene structures, chromosomal positions, syntenic analysis, gene duplication events (TD, PD, TRD, WGD and DSD) and other structural characteristics. In addition, in silico analysis and qRT-PCR expression patterns indicated that *PavMYBs* member may play a vital role in bud dormancy. Furthermore, identifying and evaluating the *PavMYB* genes involved in dormancy induction will assist to understand the dormancy processes which ultimately help to enhance the fruit production.

## Material method

### Plant materials

The sweet cherry cultivar ‘Royal Lee’ was grown at Shanghai Jiao Tong University’s experimental farm in Shanghai, China (31.25°N, 121.48°E). Gisela 6 (G6) was used as rootstock for grafting diploid cultivars. Under the same agricultural practices, all trees were planted at a 5–6 m spacing. Bud, flower and fruit sample were taken at BBCH-50, 65 and 90 respectively. Until further usage, all experimental materials were frozen in liquid nitrogen and kept at − 80 °C.

### Identification, database search and characteristics of the *MYB* genes in *P. avium*

The whole-genome sequence of *P.avium* (v1.0) along with GFF3 (General feature format file) were downloaded from GDR database (https://www.rosaceae.org/) [41]. Additionally, Arabidopsis MYB amino acid sequences were retrieved from the Arabidopsis Information Resource (TAIR) (http://www.arabidopsis.org/) and BLASTp as the protein sequence of Arabidopsis were used as query. The MYB domain’s HMM (Hidden Markov Model) profile was collected from the Pfam version 31.0 database [42]. Following that, BLASTp (E < 1e^− 5^) resemblance searches of MYB and variations of HMM builds (E-value = 1e ^− 3^) were being used to classify MYB in *P. avium*. The sequence was further analyzed by using Pfam (http://pfam.xfam.org) [42], InterPro (http://www.ebi.ac.uk/interpro/sea) [43], SMART (http://smart.emblheidelberg.de) [44] and PROSITE (https://web.expasy.org/protparam) [45]. Finally, the sequences were eliminated which omitted the central or entire MYB_DNA-binding domain. The MYB protein sequence were further subjected to the Expasy software (https://web.expasy.org/protparam/) for molecular characteristics (length of amino acids, molecular weights and isoelectric points) while CELLO2GO (http://cello.life.nctu.edu.tw/cello2go/) (E-value = 0.001) was used for analyzing the subcellular localization [46, 47].

### Phylogenetic analysis and prediction of paralogous gene pairs

All MYBs subfamily members’ amino acid sequences were matched using default parameters (pairwise deletion, 1000 bootstrap) in the multiple sequence alignment tool clustalX software. The phylogenetic tree (*P. avium* and *Arabidopsis thaliana*) was built with Molecular Evolutionary analysis (MEGA-X) by utilizing maximum likelihood method (ML-M). On the other hand, neighbor joining method (NJ-M) [47] was also used for phylogenetic tree construction between three Roseacea species (*Prunus avium, Prunus persica, Fragaria vesca,* and *Prunus mume*) [48, 49]. Furthermore, the itols program (http://itol.embl.de) was used to visualize phylogenetic trees [50]. The resultant tree was used to determine the evolutionary origins of MYBs. The paralogous gene pairs (PGPs) were characterized from the final tree as proteins that were exist in pairs on the terminal nodes, well defined by their bootstrap values.

### Duplication of MYB genes, ka/ks analysis and collinearity relationships

The MCScanX programme was used to conduct interspecies and intra-species collinearity analyses at the protein with an E-value of 1e-5 [51]. The duplicate gene classifier script in the MCScanX program was used to quantify the various forms of duplications (tandem, dispersed, WGD or segmental and proximal duplication) [51]. When the pairs of genes inside two segmental regions have cointegration, WGD or segmental duplication gene pairs were assumed. We referred to gene pairs with two duplicated genes as tandem duplications when they were sequential. Collinearity relationships and gene duplications were envisioned by means of circos software and TBtools [52, 53]. Synonymous mutation rates (ks; mutations/substitutions that outcome in a single amino acid alteration on a specified polypeptide) and the non-synonymous (ka mutations/substitutions that would not lead in an amino acid sequence change) for subsequent duplication gene pairs were easily extracted from the Plant Genome Duplication Database (http://chibba.agtec.uga.edu/) [54]. The Ks values were utilized to compute the estimated date of the duplication event (T = Ks/2 k) [55], assuming clock-like rates (k) of synonymous substitution of 6.96 * 9 10^− 9^ substitutions/synonymous site/year for sweet cherry. MAFFT software was used for alignments and ka/ks calculators were used to compute the ka/ks ratio within each duplicated gene pair and multiplealignments(https://github.com/qiaoxin/Scripts_for_GB/tree/master/calculate_Ka_Ks_pipeline) [56].

### Intron-exon, conserved motif and domain analysis

For the detection of conserved domains of each gene, we used the HMMER version 3.1 programme (http://hmmer.org/) and CDART (Conserved Domain Architecture Retrieval web Tool) (https://www.ncbi.nlm.nih.gov/Structure/lexington/lexington.cgi) to search against the Pfam database version 31.0 [57]. The Gene Structure and Display server v2.0 (http://gsds.gao-lab.org/) was utilized to envision the next major features: location and arrangement of intron-exon, conserved elements and binding sites [58]. The conserved motifs were analyzed through the Motif Elicitation (MEME) software (http://meme-suit.org) [21].

### Chromosomal localization analysis

The starting and ending positions of each of the defined MYB genes were obtained from the Plant Transcription Factor Database and validated using the GFF3 file. CLC sequence viewer version 7 (CLC Bio, QIAGEN A/S, Aarhus, Denmark) was used to verify these locations along chromosomes of the downloaded Rosaceae genome (httpp://www.rosaceae.org/). Finally, Mapinspect software (https://mapinspect.software.informer.com/) [47, 59, 60] was being used to analyze the data.

### Transcriptomic analysis

The NCBI SRA database (http://www.ncbi.nlm.nih.gov) was used to obtain RNA-seq data of *P. avium* on different dormancy stages (organogenesis, paradormancy, endodormancy, ecodormancy and dormancy release) through following SRR8984342, SRR8984344, SRR8984360, SRR8984359, SRR8984367, SRR8984366, SRR8984382, SRR8984381, SRR8984403 and SRR8984402 accession numbers. Data from the SRA database was first encrypted into the FASTQ format using the SRA toolkit. With default parameters, the Hisat2 programme was used to map each dataset to the reference genome. The expression level was computed by using StringTie programme and TPM (Transcripts per Kilobase Million) values were obtained from RNA-seq data. Finally, heat maps were generated through TBtools [61].

### RNA isolation and quantitative real time PCR

The expression patterns of *PavMYB’s* gene was examined by using qRT-PCR. Total RNA was extracted from bud, flower and fruit by using RNAprep pure plant kit (Tiangen). The RNA (1 μg) was utilized to execute reverse transcription was done through the PrimeScript™ RT reagent Kit with gDNA Eraser (Takara). Specific primers was designed and checked through Beacon designer 7.9 software and Actin was used as reference gene (Table S2). Three biological with three technical replicates were used. The quantitative real-time qRT-PCR was done with each reaction consisted of 1 mg of RNA. The qRT-PCR system consisted of 10 mL SYBR Premix Ex TaqTM II, 2 mL cDNA, 6.4 mL water, and 0.8 mL forward primer and reverse primer under the following conditions: 50 °C for 2 min, 95 °C for the 30s, followed by 40 cycles of 95 °C for 15 s, 60 °C for 20s and 72 °C for 20s 4°C for ∞. The 2^-ΔΔct^ method was utilized to compute the relative expression levels of *PavMYB* genes [62].

## Results

### Identification and molecular characterization of MYB gene family in sweet cherry

Genome-wide analysis was performed to identify the R2R3-MYB genes in *P. avium* genome. The MYB HMM profile (Pfam: 00249) was being utilized as a query in a BlastP search against the sweet cherry genome database. Finally, 69 *PavMYBs* proteins were identified and their sequences were further examined for the presence of MYB_DNA-binding domains by using the Pfam, Interpro tool, and SMART database. Single MYB DNA-binding domain was identified in 51 *PavMYB* genes, 14 *PavMYB* genes contained two domain and 2 *PavMYB* genes had four domains. *Pav_sc0000800.1_g1130.1.mk* was the only candidate gene which contained 3 domains. (Table S1). In meanwhile, we discovered that *PavMYB* genes from the same subfamily having identical domain distributions and compositions elaborated that these subfamilies had a similar evolutionary history. *PavMYB* proteins had amino acid lengths ranging from 63 bp to 1056 bp with average of 272 bp (Table S1). Moreover, the molecular weight of *PavMYB* genes varied from 7421.6 to 117,470 kDa with an average of 30,452.16 kDa and the approximate Isoelectric point (pI) ranged from 4.09 to 10.44 with an average of 6.78 (Table S1).

### Phylogenetic relationship of the sweet cherry MYB gene family

**Fig. 1:**
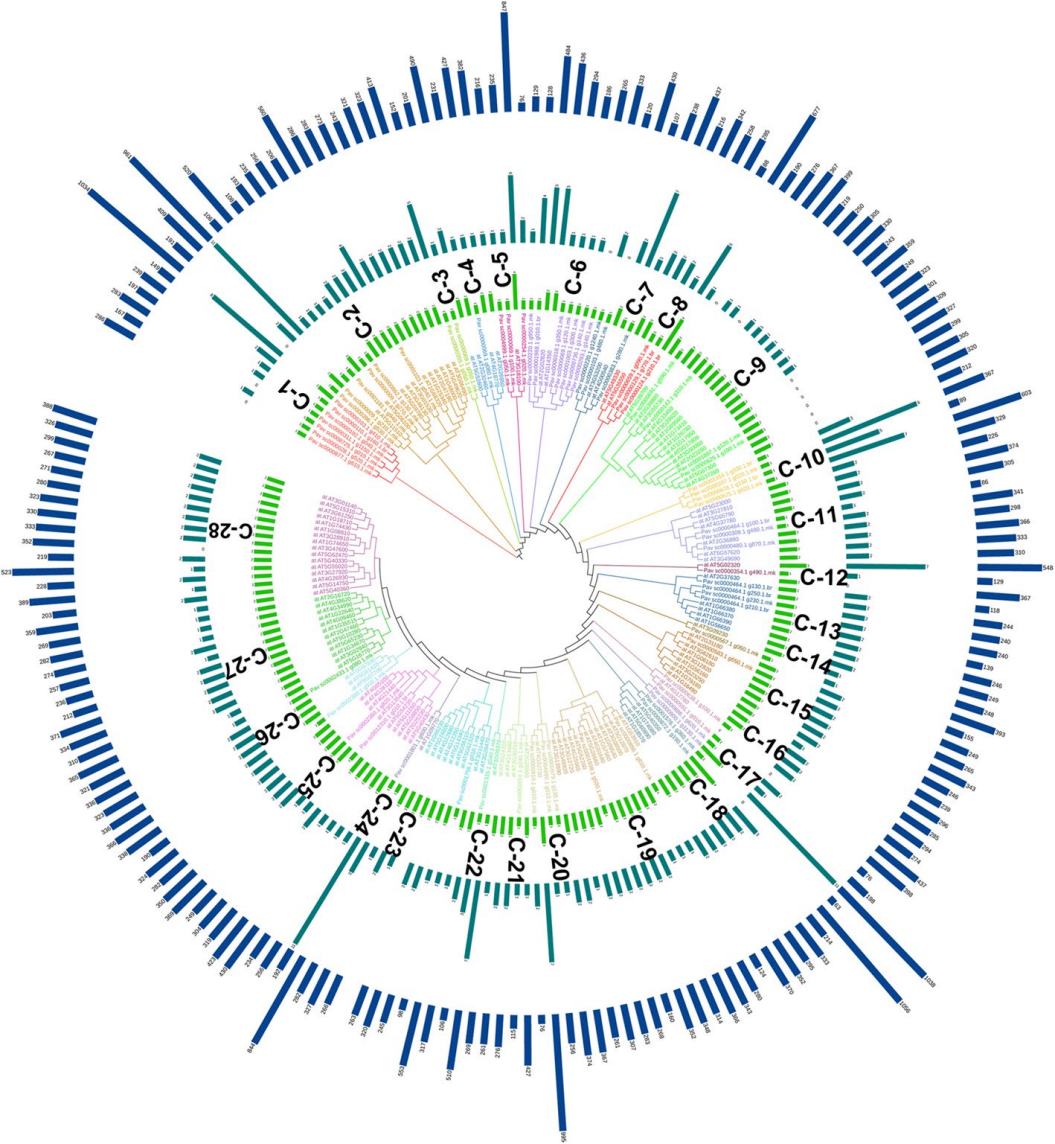
Phylogenetic tree of MYB protein *P. avium,* and *A. thaliana* with each color representing a subfamily (C1-C28) of MYB genes. Green, teal and blue bars indicate domain, number of introns and length of amino acids, respectively

To investigate the evolutionary relationship of the MYB genes in sweet cherry, a maximum likelihood method (ML-M) phylogenetic tree were constructed by using the full-length R2R3-MYB protein sequences from *P. avium* and *Arabidopsis thaliana*. We classified the R2R3-MYB members of sweet cherry into 28 subgroups (C1-C28) according to the sequence similarity and topology with Arabidopsis (Fig. 1). Further, the evolutionary relationship was examined among the four Rosacea species. Moreover, we used two phylogenetic inference methods with bootstrapping 1000 times, namely maximum likelihood method (ML-M) with *P. avium* and Arabidopsis while, neighbor-joining method (NJ-M) with (*P. persica, F. vesca, P. mume, and P. avium*) to visualized phylogenetic trees to justify the topologies. These two trees explained similar topologies because the ML-M tree has higher bootstrap values than the other phylogenetic tree, which was the reason we had chosen the ML-M tree for our future study (Fig. 1 and Fig. S3).

The defined clades in Arabidopsis were labeled in the evolutionary tree. Subfamily C9 contained the highest MYB members (18), while the subfamily C3, C12, C14 and C24 had the lowest two MYB members (Fig. 1). MYB protein sequences from sweet cherry contributed in all subfamilies with Arabidopsis except C1 subfamily. Our results proposed that they may have been gene gained or lost events that happened through the evolutionary process.

### Conserved motifs, domain and intron number evaluation

**Fig. 2:**
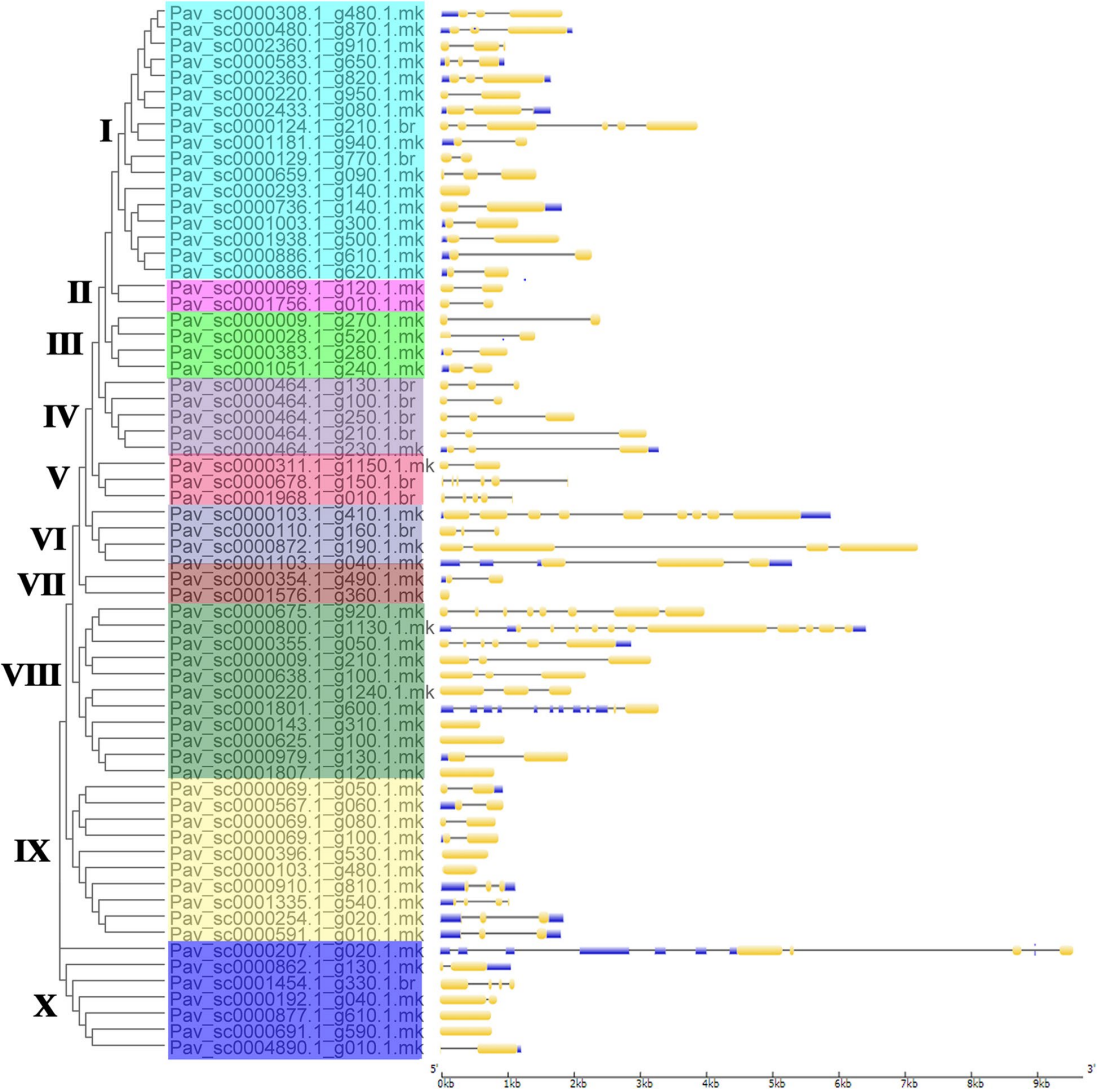
Phylogenetic relationships and gene structures (introns/exons) *MYB* genes of the sweet cherry. The gray line represented the intron (s) while yellow and blue color illustrated the exon (s) and UTR (s)

**Fig. 3:**
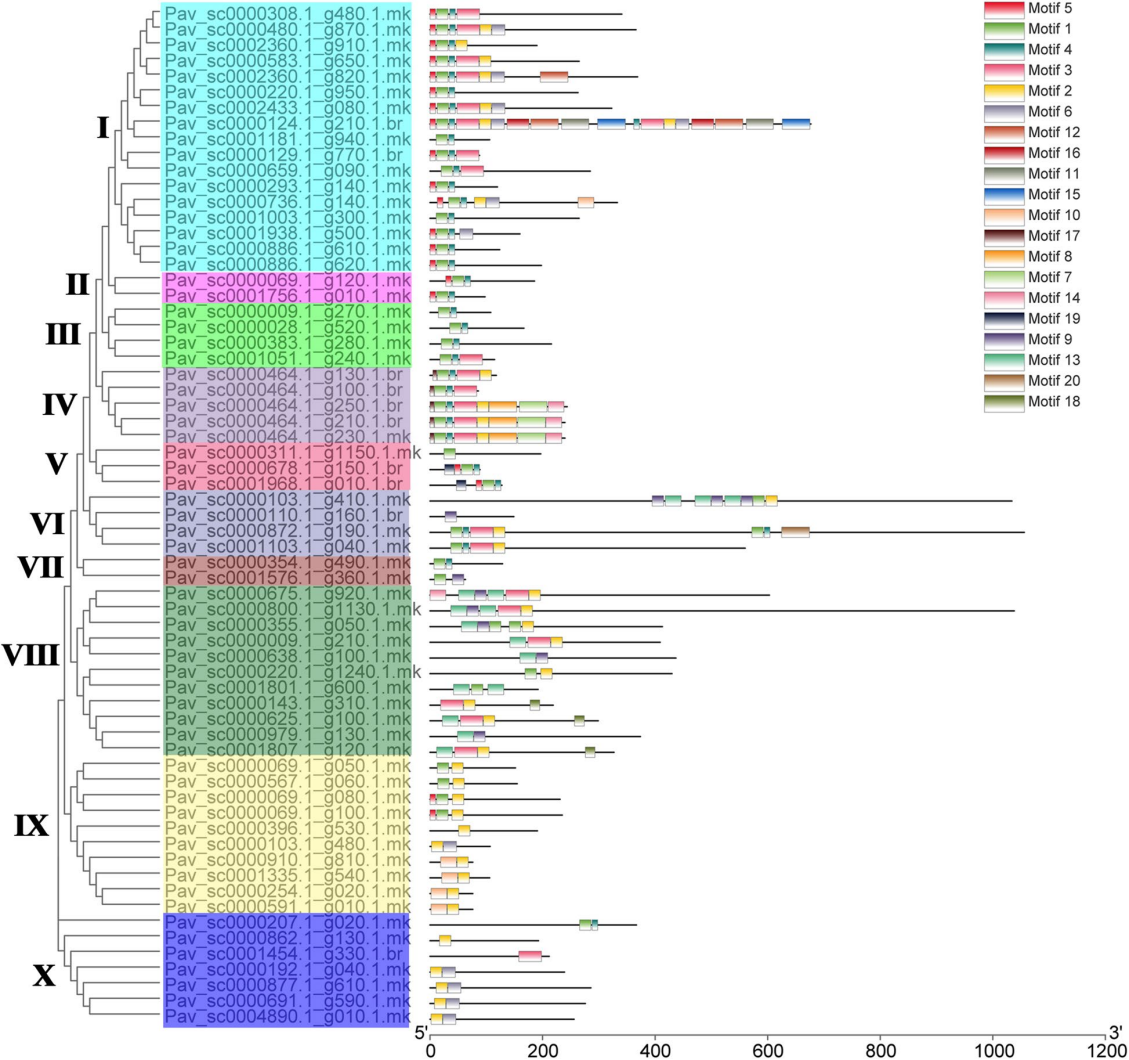
The phylogeny and conserved motifs in the *MYB* TFs in *Prunus avium* and figure legends are mentioned on the top

A total of 69 MYB proteins from *P. avium* were checked against the Pfam database to validate that they contain the MYB DNA-binding domain. Each MYB gene’s protein domains were determined (Fig. 1 and Table S1). At least one MYB DNA-binding domain was found in all MYB genes that encode proteins, while 14 genes encoded proteins with two MYB DNA-binding domains such as *Pav_sc0000480.1_g870.1.mk, Pav_sc0000464.1_g250.1.br* and *Pav_sc0000143.1_g310.1.mk*. Two genes including *Pav_sc0000124.1_g210.1.br*, *Pav_sc0000872.1_g190.1.mk* contained four MYB DNA-binding domains while only *Pav_sc0000800.1_g1130.1.mk* had 3 MYB DNA-binding domains (Fig. 1 and Table S1). We revealed that MYB genes from the same subfamily had equal domain distributions and compositions, implying that these subfamilies had a same evolutionary history. The position of introns was retained throughout evolution and might be implemented to determine a gene’s phylogenetic relationship [63, 64]. We performed gene structure (intron/exon) analysis for further genesis of MYB gene family in sweet cherry. Most genes in the same cluster had common exon/intron structures, especially in terms of the number of introns, like I, II, III, and so on (Fig. 2). Nevertheless, there were a few notable exceptions. The members of V and VI, for example, had varying numbers of introns. Intron/exon numbers ranged from 1 to 11 in sweet cherry (Fig. 2 and Table S1). Current findings suggested that the *PavMYB* subfamily has a lot of strongly conserved structures with in subfamilies while a lot of sequence diversity between subfamilies was observed. Genomic and CDS sequences were evaluated in order to investigate the structural diversity of MYB genes. The majority of MYB genes were found in clade “I” which contained 1 to 2 introns, while two members was found in clade “II,” who has only intron (Fig. 2). Furthermore, the number of intron varied from 1 to 11, the clade “VIII” having the most (1 to 11) accordingly (Fig. 2). These findings suggested that it intron loss or gain happened during the evolutionary process of the MYB genes family. The conserved motifs were identified by using MEME web server to evaluate the sequence characteristics of MYB members. Finally, each comparison revealed twenty motifs, which were classified into 1 to 20 (Fig. 3, Table S8). The majority of MYB genes, as specified in Fig. 3, included several motifs (1, 2, 3, 4, and 5). Clade II particularly, had only three motifs (1, 4 and 5), while clade III members contained 2 motif (1, 4) but other clades had several (Fig. 3). The motif compositions of most highly associated identical MYB genes indicating that MYB genes within the same subfamily have some functional similarities. Furthermore, we discovered a subfamily-specific motif that could play a key role in subfamily-specific activities. Moreover, several motifs, such as motifs 1and 2, were identified in practically all subfamily, suggesting that these motifs are crucial for MYB gene expression with similar functions. The evolutionary study of the MYB gene family was supported by the homogeneity in the compositions of motif and intron structure of MYB genes within the same group, whereas differences between distinct groups indicated their special functionalities.

### *Cis*-acting element analysis

**Fig. 4:**
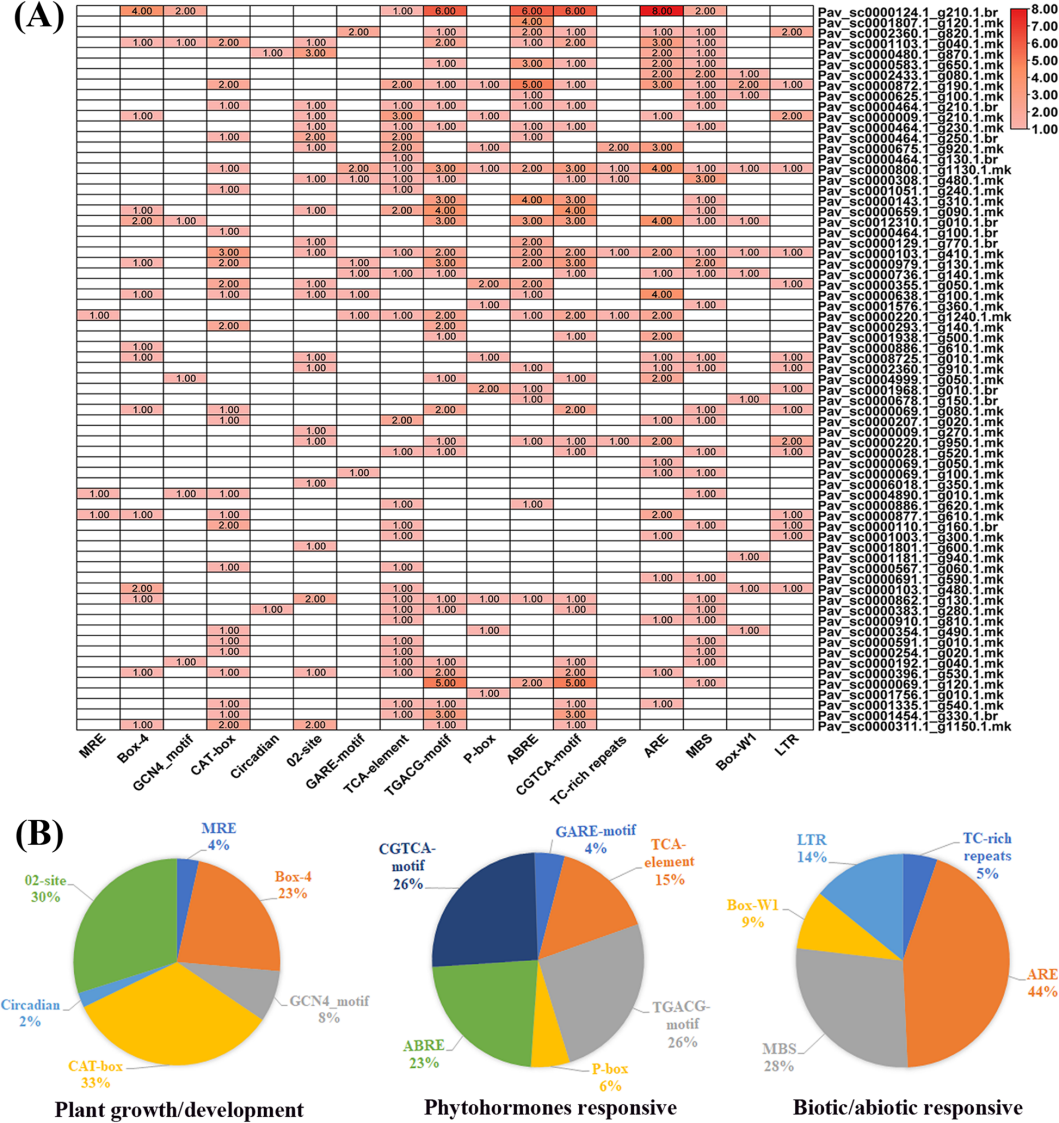
Putative *cis*-acting elements in *PavMYB* promoters using the PlantCARE database. **A** The different colors represent the number of each cis-acting elements. **B** Pie charts size represented the percentage of promoter element in each category such as phytohormones responsive, biotic/abiotic stress and plant growth & development

Two complementary regulatory components exist in the plant transcriptional mechanism: (1) trans-acting elements (2) *cis*-acting elements. Trans-acting factors are transcription factors as well as other DNA-binding proteins that attach to particular regions in *cis*-acting elements to boost or repress gene transcription. The DNA sequence in the non-coding or coding regions of the genome are defined as *cis*-acting elements. *Cis*-regulatory elements play an important role in the management of regulatory networks, particularly multi-stimulus responsive genes and defining the stress-responsive expression patterns or tissue-specific expression patterns of genes was deeply linked with *cis*-elements in their promoter regions. The *cis*-acting elements on the promoter regions were classified into three major classes: phytohormones responses, biotic/abiotic stress responses and plant growth/development (Table S3). Moreover, growth and development, *cis*-acting elements are prevalent in promoter regions, containing the Skn-1___motif, GCN4_motif, MRE, Box-4, CAT-box, O_2_-site and circadian (Fig. 4A, B). We identified the CAT-box motifs who encompassed the highest proportion (33%), which are useful for meristem expression, followed by the O_2_-site (30%) and Box-4 (23%) (Fig. 4B) that are responsible for zein metabolic control and plant growth in response to light respectively [65]. The TGA-element is engaged in auxin sensitivity, GARE-motif and P-box for gibberellin response and ERE for ethylene responsive expression. The most prevalent *cis*-element in the second group was CGTCA motifs and a TGACG _motif *cis* acting elements associated to methyl jasmonic acid (MeJA) responsiveness [66–68]. The phytohormones response related cis-elements like ABRE (23%), P-box (6%), TGACG-motif (26%), TCA-element (15%), GARE motif (4%) and CGTCA-motif (26%) were also revealed (Fig. 4B), which are linked with SA, ABA, ethylene and MeJA responses. Numerous stress-responsive elements were determined, such as ARE (44%), LTR (14%), MBS (28%), which are associated to light stress, cold and drought stress response respectively. These findings suggested that members of MYB gene family have the ability to improve cold stress response.

### Gene duplication events, Syntenic analysis and expansion patterns of the sweet cherry MYB genes

**Fig. 5:**
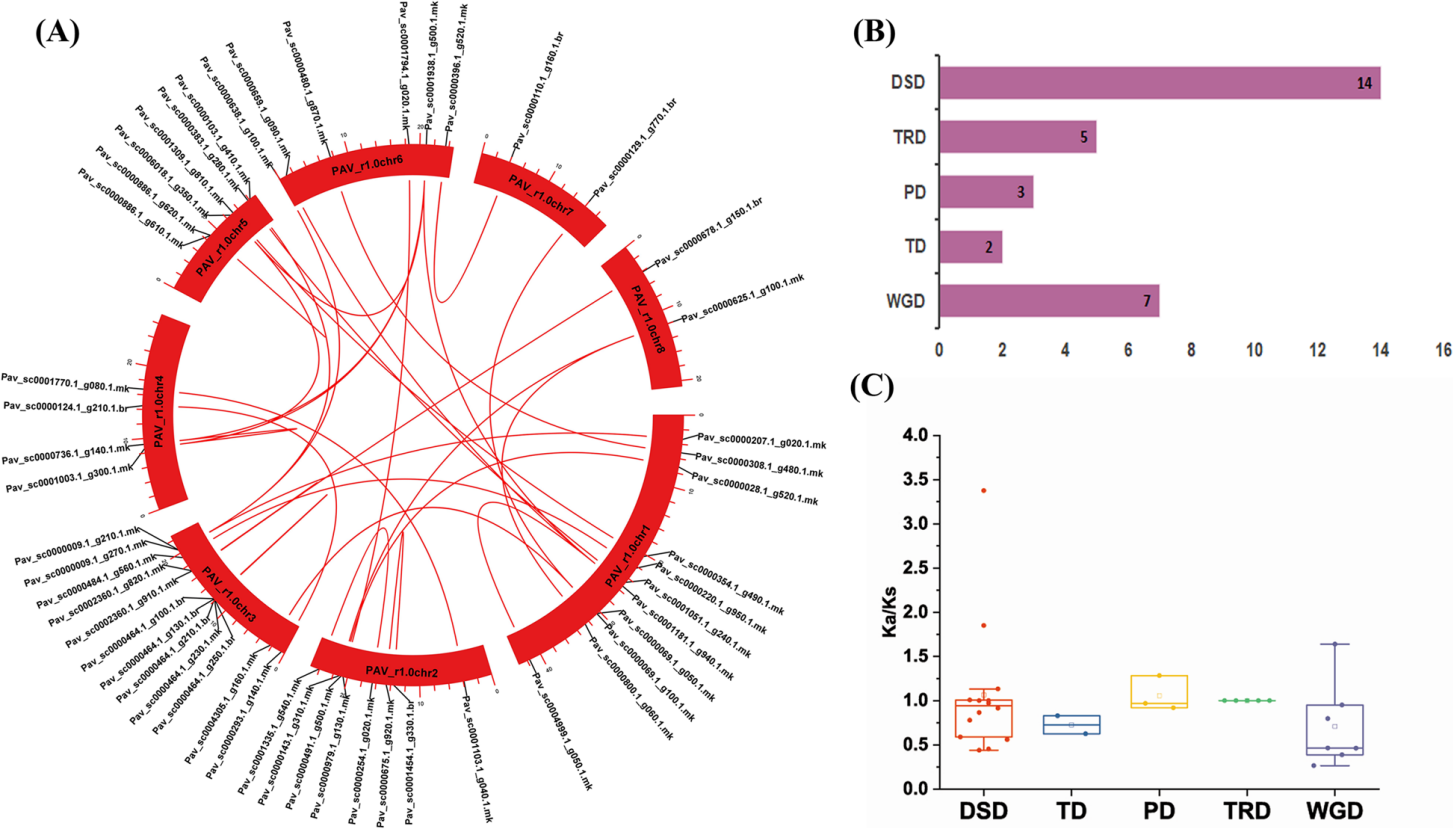
Gene duplication (DSD, WGD, PD, TRD and TD) events and localization of PavMYB genes family in *P. avium*. **A** Duplicated gene pairs placed on the different chromosomes and linked with red lines (**B**) different numbers represents the number of duplications in each event (**C**) calculated ka/ks values present the diverse gene duplication (DSD, TD, PD, TRD and WGD) events

**Fig. 6:**
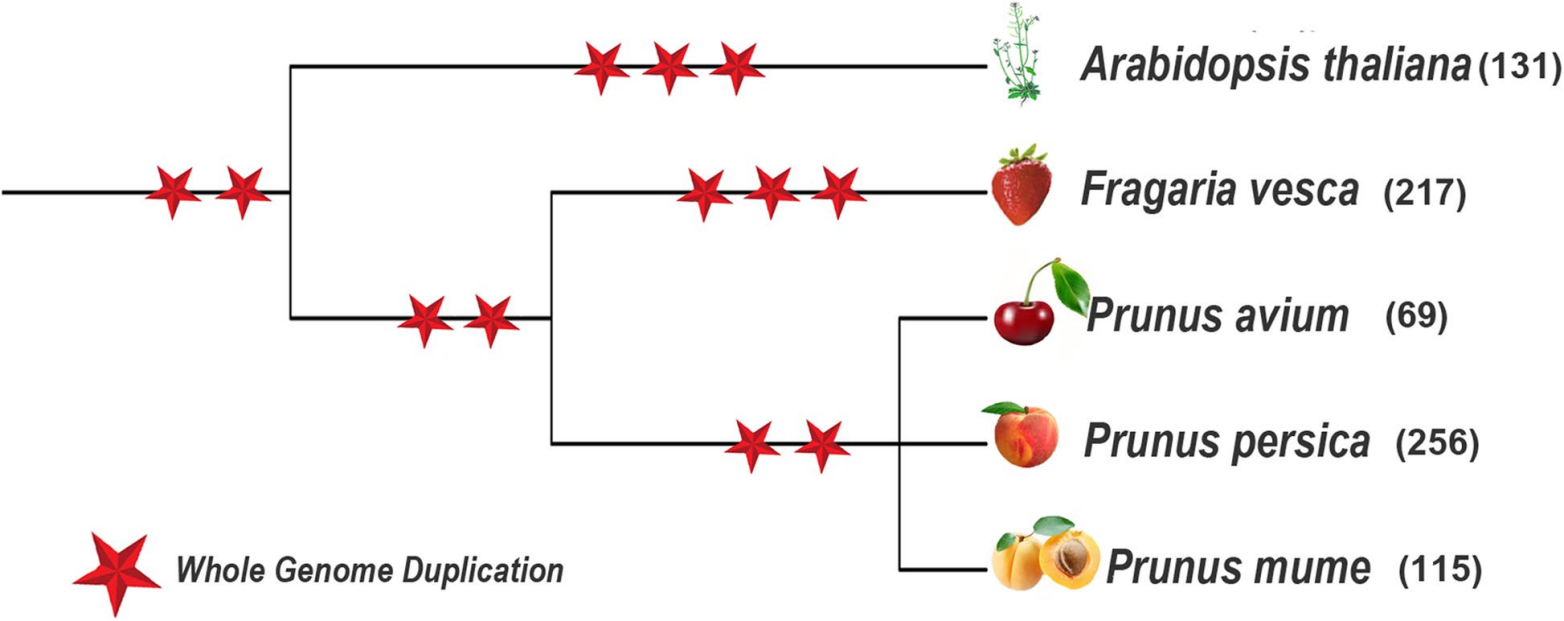
The phylogeny of the *Fragaria vesca, Prunus avium, Prunus persica, Prunus mume and Arabidopsis thaliana* genomes. The red star indicated whole genome duplication (WGD) events and the number in the parentheses indicates the total number of MYB members in each species

Expansion of Gene family and the development of novel functionalities are known to be aided by gene duplication (tandem and segmental) and divergence [68]. Duplicated genes generally mutate to acquire new functions or to divide ancestral gene functions which is essential for plant adaptation [69, 70]. An ancient whole-genome duplication (WGD) event occurred around 140 million years ago in apple [71]. We used MCScanX and circos software to visualize duplications within the genome of sweet cherry genome to evaluate the effects of duplication on the sweet cherry MYB genes family. Furthermore, *PavMYB* genes, we revealed 14 dispersed duplication pairs, 7 WGD duplication pair, 5 transposed duplication, 3 proximal duplication and two tandem duplication pairs (Fig. 5A, B, Table S5). This indicates that DSDs and WGDs play important role for MYB gene family expansion. These results revealed that duplications events contribute vital role in MYB gene expansion (Fig. 6). Moreover, DSDs and WGD duplications happened with higher frequency for expansion.

**Fig. 7:**
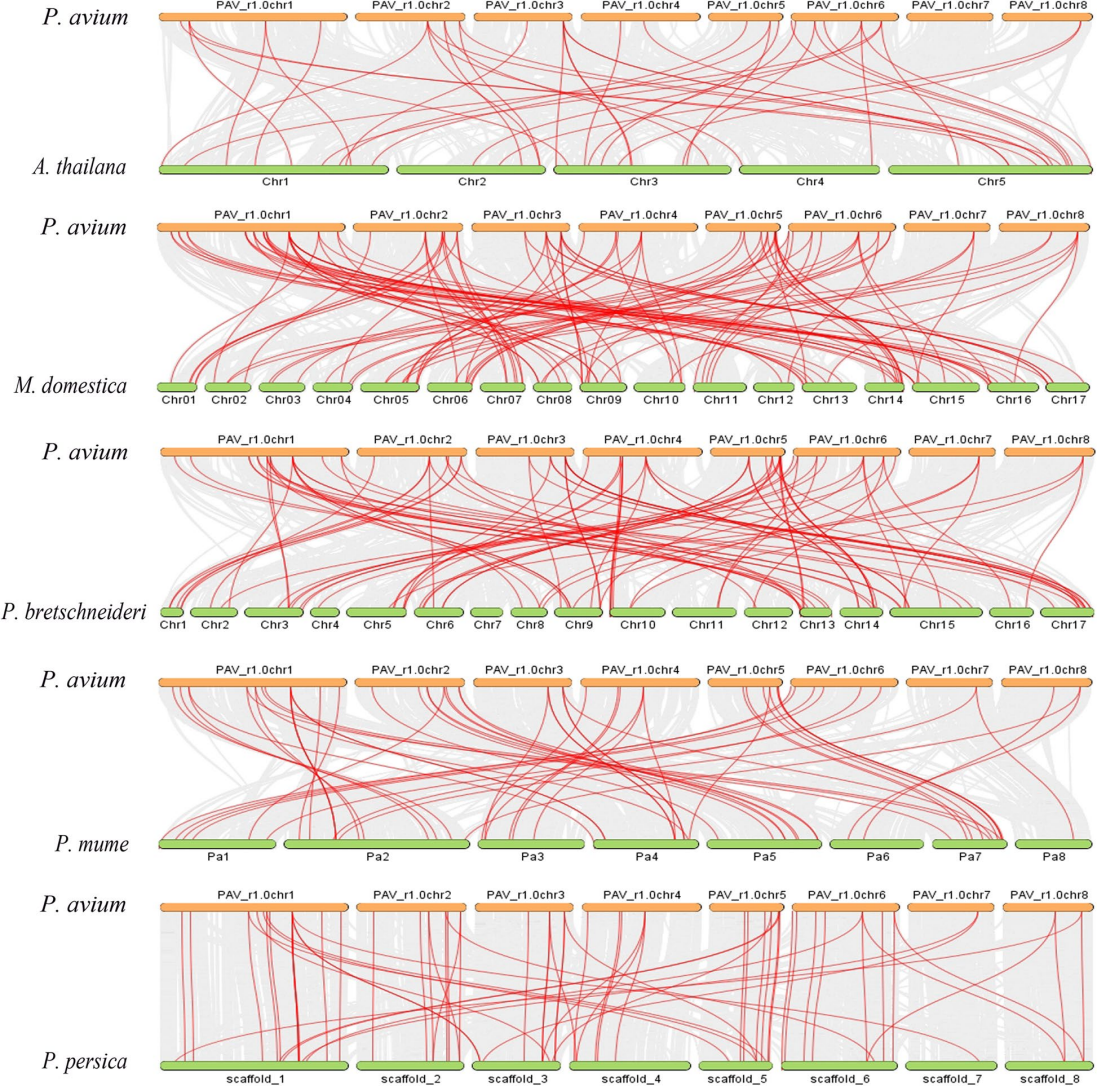
Syntenic relationship of MYB genes in *P. avium* with*, A. thaliana, M. domestica, P. bretschneideri, P. mume* and *P. persica* and syntenic genes pairs are connected with red color lines

To further understand the evolutionary relationship of MYB members in different plant species, the syntenic relationship was traced between *PavMYB’s* and homologous in other species including *P.mume, M. domoestica, P. persica, P. bretschneideri* and *A. thaliana.* These four plants belong to the Rosaceae family and shared a similar ancient. Consequently, the collinearity relationships were also analyzed between all MYB genes in four Rosaceae genome (Table S4). Total 359 collinear genes pair events were found between four Rosaceae species along with *A. thaliana*, included 57 orthologous pairs among sweet cherry and Japanese apricot, 106 orthologous pairs among sweet cherry and apple, 65 orthologous pairs among sweet cherry and peach, 91 orthologous pairs among sweet cherry and Chinese pear, and 40 orthologous pairs sweet cherry and *A. thaliana* (Fig. 7) suggesting the closer relationship among four *Rosaceae* genomes (Fig. 7). These results recommend that there are collinearity connections between the sweet cherry and the other Rosaceae genomes, signifying a potential evolutionary process between them.

For better visualizing the evolutionary history, Ka/Ks-values was calculated to estimate evolutionary evidence of duplication occurrences in general (whole genome duplication or segmental duplication). Previous research has also been shown that the apple genome was formed by two phases of ancient whole-genome duplications around 140 million years ago (Ks 1.5–1.8) and a recent whole-genome duplication around 30–45 million years ago (Ks 0.15–0.35) [72]. The Ks values were determined to investigate whole or segmental genome duplication events with in MYB gene family (Table S5). The Ks values of duplicated gene pairs were determined. In DSD, Ks value ranges from 0.5875 to 1.6836 which revealed that duplications events might well happened from 32.28 million years ago (Mya) to 92.50 Mya (Table S5). Furthermore, the Ka/Ks ratios are commonly utilized to reflect gene selection pressure and rate of evolution. Positive selection with faster evolution is indicated by Ka/Ks > 1, purifying selection with the functional constraint is indicated by Ka/Ks < 1, and Ka/Ks = 1 shows that the genes are migrating naturally [73]. In sweet cherry, the Ka/Ks ratios of duplicated gene pairs < 1 (*Pav_sc0000625.1_g100.1.mk, Pav_sc0000464.1_g250.1.br, Pav_sc0002360.1_g910.1.mk, Pav_sc0001051.1_g240.1.mk*) suggesting that MYB genes developed beneath severe purifying selection (Table S5). Moreover, in sweet cherry gene pairs like *Pav_sc0000103.1_g410.1.mk* (Ka/Ks ~ 3.37), *Pav_sc0001938.1_g500.1.mk* (Ka/Ks ~ 1.13), *Pav_sc0001181.1_g940.1.mk* (Ka/Ks ~ 1.85) *Pav_sc0000464.1_g130.1.br* (Ka/Ks ~ 1.28) and *Pav_sc0000069.1_g100.1.mk* (Ka/Ks ~ 1.63) had higher Ka/Ks ratios implying that this family might have a complex evolutionary history. For sweet cherry, the mean Ka/Ks calculated values for the TD, TRD, WGD, PD, and DSD gene pairs, which were 0.72, 1.05, 1.00, 0.70 and1.06, correspondingly (Fig. 5C, Table S5). The DSD and PD duplication events had a higher Ka/Ks ratio as compared to other mode of duplications, representing that that MYB genes have expansion and complicated evolutionary history (Fig. 5C). Positive selection occurs in certain sections of protein-coding genes at the same time, implying the emergence of novel gene functions. Strongest evolutionary constraints were engaged in the evolution history of the MYB gene which may contribute to gene function stability.

### Chromosomal distribution analysis

We construct a map of chromosomal locations based on the genomic information of sweet cherry to illustrate the dispersal of the MYB members throughout the chromosomes (Table S3). The MYB genes were found in a random pattern on 8 chromosomes, with the majority of them clustered near the tail intermediate end of a single chromosome. The highest MYB genes (15%) were found on Chr1, while, 14% were found on Chr2 and Chr3 which were organized into gene clusters (Fig. S1). Furthermore, chromosome 5 and 6 have 11.5% MYB genes, while chromosome 4 had 5% of total MYB genes. Moreover, the least MYB genes (2.8%) were found on chromosomes 8. Furthermore, on chromosomes 7, 5% MYB genes was traced and 13% genes were found on scaffold (Fig. S1).

### Go annotation analysis

**Fig. 8:**
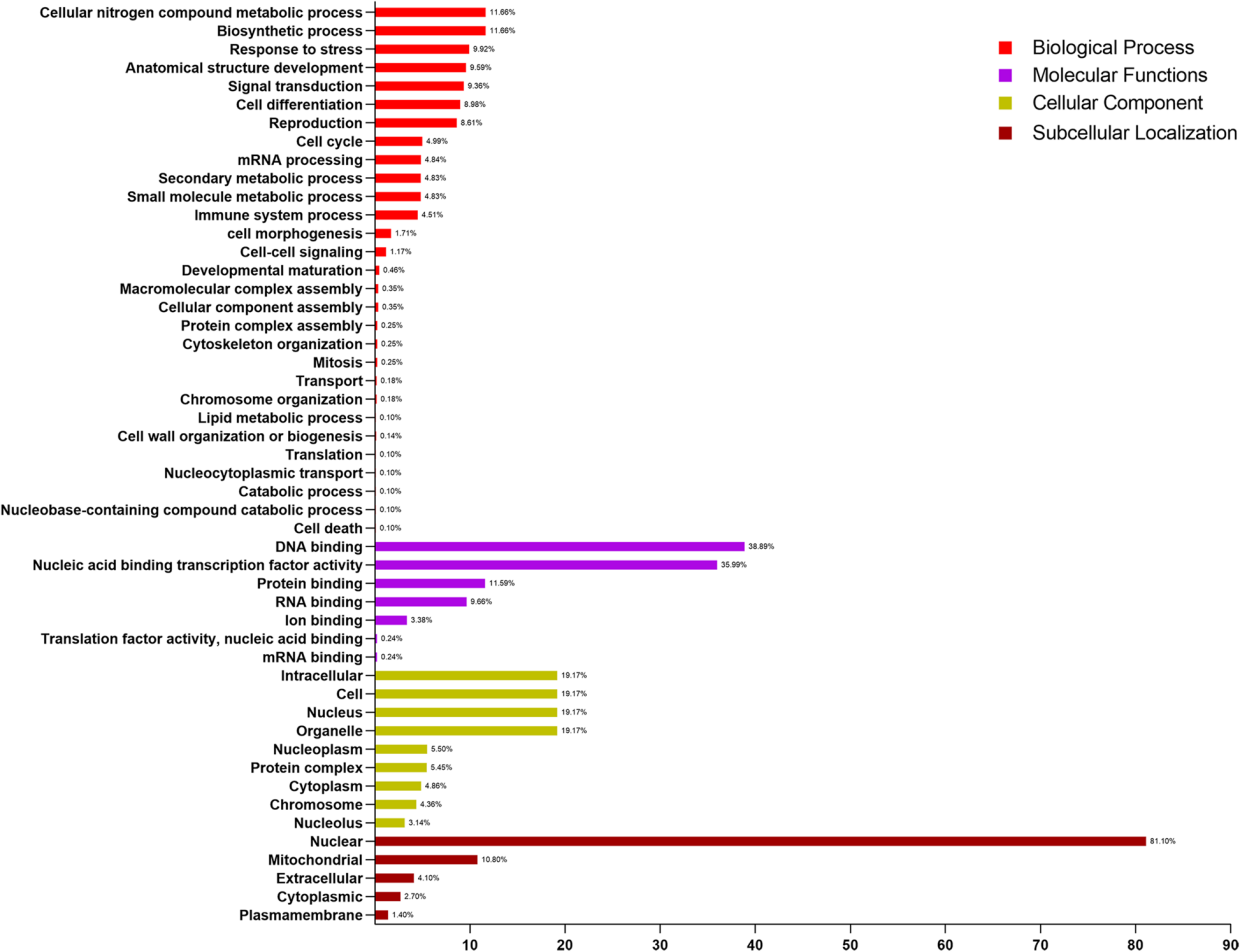
Predicted biological process, molecular functions, cellular components and subcellular localization of PavMYB gene family

Go ontology annotation analysis was performed to predict subcellular localization and evaluated different functions of MYB protein in sweet cherry. Sixty-nine *PavMYB* proteins were categorized into 50 functional groups based on protein sequence similarities and categorized into four main ontologies, namely Biological process, Molecular functions, cellular component and subcellular localization (Table S6). In the molecular process, we analyzed that more than 38.89% of annotated proteins functions in the activity of DNA binding, followed by nucleic acid binding (35.99%), protein-binding (11.59%) and ion binding (3.38%) (Fig. 8). In the biological process, *PavMYB* members involved in the cellular nitrogen compound metabolic processes (11.66%) and biosynthetic processes (11.66%), stress response process (9.92%) followed by anatomical structure development(9.59%), signal transduction (9.36%), cell differentiation (8.98%) and reproduction (8.61%) (Fig. 8). Subsequently, predicted *PavMYB* proteins are also annotated with cell cycle (4.99%), immune system process (4.51%), and cell morphogenesis (> 1%) in biological process annotation. The cellular component annotations showed that the *PavMYB* proteins annotated with the intracellular, nucleus, organelles and having the same percentage (19.17%) and nucleoplasm (5.50%) (Fig. 8). Furthermore, in subcellular localization, these results showed that highest percentage of the *PavMYB* proteins were observed in nuclear with 81.10% while the mitochondrial, extracellular, cytoplasmic and plasma membrane contained 10.80, 4.10, 2.70 and 1.40% respectively.

### Transcriptomic analysis

**Fig. 9:**
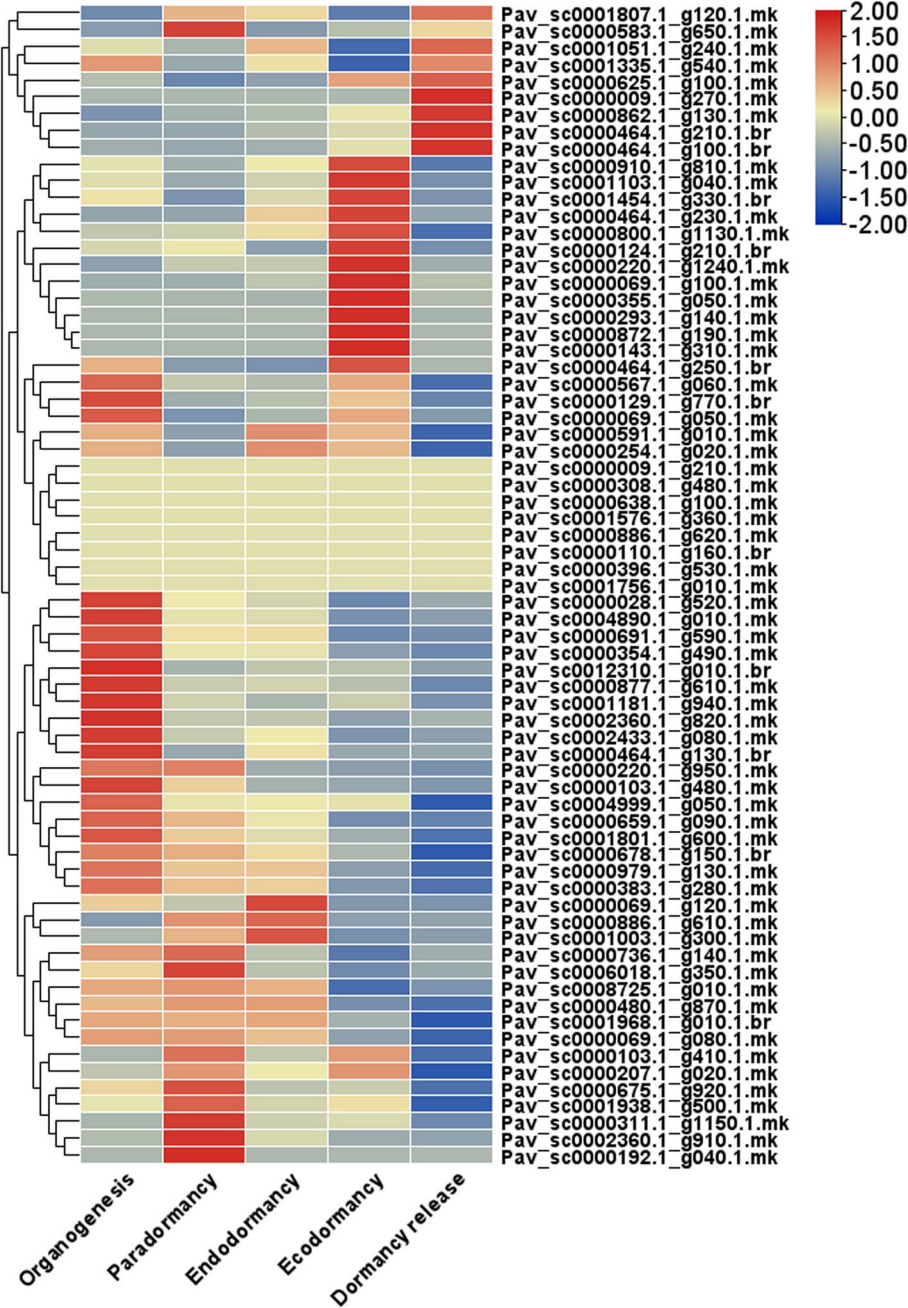
Heat map of *PavMYB* genes expression in different dormancy related phases data (organogenesis, paradormancy, endodormancy, ecodormancy and dormancy release). Blue, violet and red color indicated no expression, low expression and high expression respectively

RNA-seq datasets were used to assess the expression patterns of the MYB genes in different phases of dormancy under normal growth conditions. The amount of Transcripts Per Kilobase Million (TPM) were counted and compared across the investigated samples to obtain standardized RNA-seq data (Table S7), which are visualized in form of heat map (Fig. 9). Based on the current findings, MYB genes showed significant expression patterns on particular stage as shown in (Fig. 9). According to RNA-seq data MYB genes showed three types of expression level, each of which had genes that were predominantly expressed during distinct phases of bud dormancy (Fig. 9). The expression of nine MYB genes (*Pav_sc0001807.1_g120.1.mk, Pav_sc0000659.1_g090.1.mk, Pav_sc0001968.1_g010.1.br, Pav_sc0000678.1_g150.1.br, Pav_sc0000028.1_g520.1.mk, Pav_sc0000877.1_g610.1.mk, Pav_sc0001181.1_g940.1.mk, Pav_sc0000910.1_g810.1.mk* and *Pav_sc0000354.1_g490.1.mk*) were considerably higher across all stages of dormancy. The findings also revealed that *PavMYB* genes were seldom expressed in paradormancy than in endo dormancy (Fig. 9). In all dormancy phases, majority of genes were showed highest expression level, this phenomenon revealed that these genes play key role for dormancy regulation. During data analyzing it was also found that some genes (*Pav_sc0000009.1_g210.1.mk, Pav_sc0000464.1_g230.1.mk, Pav_sc0000308.1_g480.1.mk, Pav_sc0000638.1_g100.1.mk, Pav_sc0001576.1_g360.1.mk, Pav_sc0000886.1_g620.1.mk, Pav_sc0000110.1_g160.1.br, Pav_sc0000192.1_g040.1.mk, Pav_sc0000396.1_g530.1.mk, Pav_sc0001756.1_g010.1.mk*) were remain silent during all dormancy stages (Fig. 9). This phenomenon demonstrated that these genes are not involved in the dormancy initiation or dormancy elimination. These group of genes might be involve some other important functions in later stages of floral development in sweet cherry. Some genes like *Pav_sc0000009.1_g270.1.mk* remain silent in whole dormancy phases but show the aggression at dormancy release while some other genes like *Pav_sc0000872.1_g190.1.mk* and *Pav_sc0000143.1_g310.1.mk* had indicated peak expression in ecodormancy phase. These results illustrated that *PavMYB* genes also had stage specific expression patterns too.

### Expression analysis

**Fig. 10:**
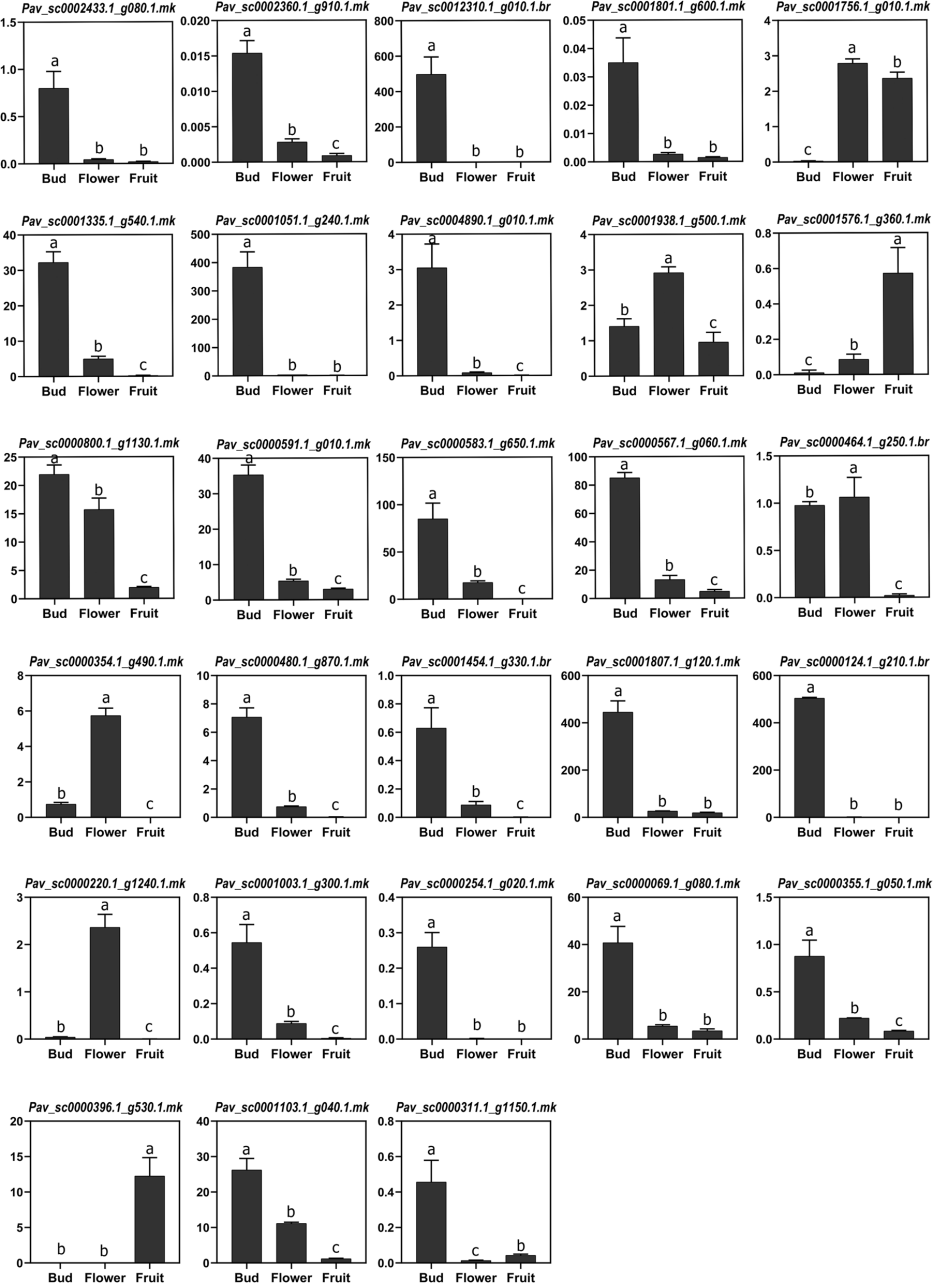
Relative expression patterns of *PavMYB* genes (C1-C28) through qRT-PCR on different tissues (bud, flower and fruit) in sweet cherry. Different letters indicate at 0.005 probability level (t-test). Mean ± SE of three biological replicates (each having 3 technical replicates)

Moreover, for further investigate the expressional behavior of *PavMYB’s* in dormancy and in other later developmental stages, we chose 28 genes (one gene from each clade) to explore the expression levels in bud, flower, and fruit. The qRT-PCR results validated the RNA-seq data, indicating that these genes had different expression profiles at the bud, flower and fruit. *Pav_sc0002433.1_g080.1.mk, Pav_sc0002360.1_g910.1.mk, Pav_sc0012310.1_g010.1.br, Pav_sc0001335.1_g540.1.mk, Pav_sc0001051.1_g240.1.mk, Pav_sc0004890.1_g010.1.mk, Pav_sc0000591.1_g010.1.mk, Pav_sc0001807.1_g120.1.mk, Pav_sc0000124.1_g210.1.br, Pav_sc0000254.1_g020.1.mk and Pav_sc0000311.1_g1150.1.mk* expressions were considerably upregulated at bud stage (Fig. 10), revealing that these genes are engaged in the dormancy. *Pav_sc0001756.1_g010.1.mk, Pav_sc0000396.1_g530.1.mk*, and *Pav_sc0001576.1_g360.1.mk* expression levels were identical to RNA-seq data, their expression being elevated only at flower and fruit. No expression was observed on bud stage. Results demonstrated that these genes (*Pav_sc0001756.1_g010.1.mk, Pav_sc0000396.1_g530.1.mk, and Pav_sc0001576.1_g360.1.mk*) were remained silent in bud dormancy and show their expression in further stages. Some genes like *Pav_sc0001938.1_g500.1.mk* and *Pav_sc0000800.1_g1130.1.mk* had identical expression profiles all dormancy stages while remaining other genes had variable expression levels (Fig. 10).

## Discussion

Across all eukaryotes, MYB genes play a critical role in various functionalities. *Arabidopsis thaliana* [14], *Vitis vinifera* [74], *Oryza sativa* [14], *Eucalyptus grandis* [75], *Zea mays* [13], *Setaria italic* [76], *Solanum tuberosum* [23], *Solanum lycopersicum* [24] and *Actinidia chinensis* [25] have all presented comprehensively and systematically study of MYB gene family. The sweet cherry (*P. avium* L.) Genome Sequence Project was accomplished in 2017 [77, 78], hence the sweet cherry genome sequence might be valuable for genome-wide analysis of MYB gene family. *PavMYB* gene family was not extensively studied yet and its majority functions remain unclear so far. In this research, The *PavMYB* family was extensively studied with analysis of phylogeny, gene structures, promoter regions, gene duplication events, sequence characteristics, chromosomal locations, GO annotations and expression patterns. Sweet cherry has fewer *PavMYB* genes (69) than, *Oryza sativa* (155) [14], *Zea mays* (132) [13], *Setaria italic* (209) [76], *Malus pumila Mill* (229) [79], *Hedychium coronarium* (253) [80], *Morella rubra* (174) [81], *Populus deltoids* (152) [82] while greater than *Ginkgo biloba* (45) [83]. These results illustrated that the *R2R3-MYB* gene family expansion occurred in sweet cherry as compared to *Ginkgo biloba*. The result also demonstrated that R2R3-MYB genes replication happened in evolutionary history and played a vital role in the regulation of plant specific development [84, 85]. The loss and gain of particular MYB gene members instigated functional divergence [20, 84]. This phenomenon also revealed that certain evolutionary events happened within MYBs of different species [86] which were very helpful for gene expansion in sweet cherry.

Gene duplication are important events that play vital role in plant evolutionary history and divergence of new functions [87, 88].The expansion of gene families has been facilitated greatly by gene duplication events [89]. In this investigation, a majority of the *PavMYB* genes were identified to have dispersed and whole genome duplications. The 69 *PavMYB* had total of 31 duplication events, with the majorities involving dispersed duplication (14) and whole genome duplication (7) while in *Ananas comosus* [90], *Arabidopsis thaliana* [89], *Citrus sinensis* [91] and *Solanum tuberosum* [92] similar patterns of MYB gene duplication pairs were identified. The studies suggested that, DSDs (dispersed duplications) and WGDs (whole-genome duplications) events are crucial for the development of MYB genes family. Collinearity analysis reveals a striking similarity between syntenic orthologous groups and phylogenetic relationships. Ka/Ks can represent the selection pressure in biological evolutionary process [93]. All of the identified homologous pairs were subjected to Ka/Ks analysis, the results of 69 pure *PavMYB* replications revealed that the most of *PavMYB* gene family has experienced purifying selection, demonstrating that it has been involved in a highly conserved evolution. Moreover, positive selection has also occurred in some *PavMYBs*, which indicated that novel gene functions. Whole-genome duplication in Rosaceae species was happened about 5 billion-year-old which raised the chromosomes number up to 9 [94], possibly resulting in the massive MYB family in sweet cherry. Furthermore, 13% *PavMYBs* could not be mapped to any chromosome, which may be related to poor quality of the sweet cherry genome sequence or excessive heterozygosity.

Subsequently, to understand the *PavMYB* genes’ evolutionary relationship, the R2R3-MYB proteins were divided into C1-C28 groups based on the phylogenetic tree with Arabidopsis. The *PavR2R3-MYB* genes grouped with Arabidopsis orthologs in several branches, suggesting that the R2R3-MYB function was extensively conserved across species. The phylogenetic connections of MYB proteins in sweet cherry and Arabidopsis also revealed that mostly clades had varied numbers of *AtMYB* and *PavMYB* genes, demonstrating that these two species had evolved in a similar evolution. In Arabidopsis, two 4R-MYB genes and five 3R-MYB genes have been discovered so far [95]. Nevertheless, in the phylogenetic tree, several *PavMYB* genes from sweet cherry were not grouped with the *AtMYBs* from *A. thaliana*, signaling that such genes are not retained between sweet cherry and *A. thaliana* and that their roles may be unique. Furthermore, in the subfamilies evaluated, *PavMYBs* and *AtMYBs* were not represented equally in evaluated subfamilies. For instance, there were no *AtMYBs* in subfamily C1. These results indicate that following divergence from the most recent primitive ancestor, species-specific MYBs gene were recruited in sweet cherry or eliminated in Arabidopsis lineages.

Subsequently, our study demonstrated that, most genes in the identical group have the similar position of the exon-intron structure, nevertheless there are fewer irregularities, which may be due to gain, loss, or skimming of introns during the formation of MYB gene family [96]. Identical findings have been observed in apple [79], suggesting that evolutionary processes of MYB members within the same family may also be similar. Several genes in plants might well be eliminated all through the evolution process [97]. In the current study, numerous genes have only 1 intron (Fig. 6 and Table S1), indicating the intron loss in evolution process. The previous research illustrated that in plants during evolution, selection pressure is the source of intron gain or loss, and genes evolve themselves into several exon-intron structures to achieve typical functions [98]. The absence of introns in genes would accelerate the evolution of genes through gene copying [99]. The function of the genes without any intron and those with one intron were found to be identical in their evolution and functions. These results exhibited that these genes endure conserved under the evolution process and own highly functional similarities. Moreover, most of the *PavMYB* genes contained similar motif compositions in the identical subfamily. A total of 20 diverse motifs were identified among the MYB genes of the sweet cherry (Fig. 5), each genes containing at least two of them. The arrangement and amount of the 20 distinct motif types in sweet cherry expressed that MYB members were functionally diverse. The existence of an identical motif and intron-exon structure between genes suggested that they may have functional similarities.

The *cis*-regulatory elements analysis demonstrated that, almost all *PavR2R3-MYB* genes contained several *cis*-acting elements like ABRE TGACG-motif and CGTCA-motif, MBS and LTR etc. which are related to abiotic stresses like low temperature response, defense/ response and wound response. Previous studies illustrated that MYB regulate the ABA biosynthesis signaling which help the plants in dormancy phase under unfavorable lower temperature [100–103]. The *MYB96*-regulate alterations in ABA levels might lead to change GA metabolic activities to confirm a dormancy state in Arabidopsis [38]. Total of 14% genes contained LTR elements and 28% genes contained MBS on the promoter regions. These results also indicated that the *Pav_sc0000124.1_g210.1.br and Pav_sc0000872.1_g190.1.mk* contained ABRE elements (Fig. 3B). The expression of these genes obviously differed under cold stress. A total of 26% TGACG-motif and CGTCA-motif, 4% GARE motifs and 23% ABREs were the main *cis*-elements of gene expression under responses to dormancy. Those results indicated the potential function of *PavR2R3-MYB* genes in response to dormancy in sweet cherry. In general, the results showed that most *PavR2R3-MYB* members were involved in various response to hormone and abiotic stress responsive.

Moreover, a unique DNA-binding domain may be found in the N-termini of MYB TFs (Dubos et al., 2010) and domain consists of one to four incomplete repetition units, each of which has 3 helices. The 2nd and 3rd helices (R2and R3) normally form an HLH configuration for DNA binding. This study discovered a total of 69 *PavR2R3-MYB* proteins. The R2 repeats had three evolutionarily conserved Trp residues, but initial Trp (R3) residue was unpredictable. Substitution of the first Trp residue might result in the identification of new target genes and/or a decrease of DNA-binding ability towards target genes [104]. The N-terminal domain is rather stable, whereas the C-terminal region is more malleable [20, 97]. The *PavMYB* genes have one or more C-terminal domains and had the 20 different conserved motifs. MYB TFs containing conserved N-terminal domain and transcriptional activations or suppression C-terminal regions might play a significant role in complicated physiological processes. Moreover, mostly *PavMYBs* may be implicated in the regulation of dormancy. Gene expression patterns may deliver significant evidences for discovering the genes function. In current study, RNA-seq data was used to visualize the expression analysis on different stages of bud dormancy. We analyzed gene expression patterns at five distinct bud dormancy phases to precisely understand the complex gene expression profile of sweet cherry MYB gene family members. The *PavMYB* genes have diverse stage-specific expression patterns, which were further separated into three groups based on the expression patterns in various stages of bud dormancy (Table S7). First group contained 38 MYB members that were extensively expressed in all phases of bud dormancy. That expression pattern demonstrated that these *PavMYB* genes could have an involvement in bud dormancy. Second group contained next 23 genes had comparable reduced expression in these phases of dormancy, whereas some genes had expression that was particular to specific phase of dormancy. Eight *PavMYB* genes (out of 69) were categorized into the 3rd group that were not expressed at any kind of bud dormancy stage (Table S7). The Genes which were remain silent on dormancy stage, it might be possible that they had some others functions.

To justify the RNA-seq results, gene expression patterns can be used to deduce this essential information regarding gene activities. Recent research demonstrated MYB genes have been found to be expressed in pear tissue (bud, flower and fruit) [105, 106]. Moreover, the function of *PavMYBs* in dormancy remains unknown in sweet cherry. In order to qRT-PCR, 28 *PavMYB* genes were selected from each subfamily of phylogenetic tree for relative expression patterns in different tissues (Bud, Flower and fruit) of sweet cherry. In our current finding, most of the *PavMYB* genes were highly expressed in bud dormancy phase and 4 genes showed transcripts abundance in fruits and flowers. This phenomenon explained that MYB genes had a vital role in dormancy regulation in sweet cherry. Expression profiling of *PavMYB* genes could help us for future understand the functional characteristics of this gene family in plant growth /development and in dormancy phase. These results is based on the evidence from whole-genome analysis with bioinformatics tools, qRT-PCR and RNA-seq data revealed that *PavMYB* play vital role in bud dormancy.

### Conclusions

In this study, a comprehensive and systematic analysis was performed and identified 69 *PavMYB* genes. *PavMYB* genes were grouped into twenty eight subfamilies according to phylogenetic analysis and conserved domain. Gene duplication events were identified as the driving force for the expansion of the *PavMYB* genes family. Bioinformatics analysis was performed including, gene structure, conserved motif, physiochemical characterization, *cis*-acting elements, chromosomal localizations, transcriptomic profiling, conserved domain, synonymous and non-synonymous ratios, and collinearity relationship. Furthermore, the qRT-PCR analysis validated expression patterns estimated by RNA-seq analysis of sweet cherry floral developmental stages. Genome-wide study of MYB genes gives insights into the evolutionary history and has set a foundation for genes role and functional features, and molecular mechanism in the plant dormancy process.

## Supplementary Information

**Additional file 1: Figure S1.** Nomenclature and chromosomal locations of MYB in sweet cherry.

**Additional file 2: Figure S2.** Phylogenetic tree of MYB subfamily from *Prunus avium* and Arabidopsis. Subgroups (C1-C28) of MYB subfamilies are highlighted with different colors.

**Additional file 3: Figure S3.** Phylogenetic tree of MYB subfamily from *P. avium, F. vesca*, *P. mume*, and *P. persica*. Subgroups (I-XXVIII) of MYB subfamilies are highlighted with different colors.

**Additional file 4: Table S1.** Molecular characteristics of MYB genes in *Prunus avium.*

**Additional file 5: Table S2.** Primers for qRT-PCR.

**Additional file 6: Table S3.** *Cis*- Acting elements of MYB gene family in *Prunus avium*.

**Additional file 7: Table S4.** List of MYB orthologous gene pairs identified in *Fragaria vesca Prunus mume* and *Prunus persica.*

**Additional file 8: Table S5.** Gene duplication events and synonymous and non-synonymous ratio in *Prunus avium.*

**Additional file 9: Table S6.** Molecular process, Biological process, Cellular components and subcellular localization of MYB gene family in *Prunus avium.*

**Additional file 10: Table S7.** RNA-Seq data of Sweet Cherry on different bud dormancy stages.

**Additional file 11: Table S8.** Motif sequence in *Prunus avium.*

## Data Availability

The *Prunus avium* MYB protein sequences were collected from GDR database (https://www.rosaceae.org/). The Arabidopsis MYB protein sequences were downloaded from TAIR database (http://www.arabidopsis.org). All RNA-Seq data were downloaded from NCBI SRA database under the following accession number, SRR8984342, SRR8984344, SRR8984360, SRR8984359, SRR8984367, SRR8984366, SRR8984382, SRR8984381, SRR8984403 and SRR8984402.

## Abbreviations

MYB: Myeloblastosis
HMM: Hidden Markov Model
GO: Gene ontology
NJ: Neighbor-joining
GSDS: Gene structure display server
MEME: Multiple Em for Motif Elicitation
CDS: Coding sequence
MW: Molecular weight
pI: Isoelectric point
MCSscanX: Multiple collinearity scan toolkit
TPM: Transcripts Per Kilobase Million
gDNA: Genomic DNA
RNA: Ribonucleic acid
qRT-PCR: Real-time quantitative reverse transcription PCR
