# Supplementary figures and images for: MYB transcription factor family in sweet cherry (Prunus avium L.): genome-wide investigation, evolution, structure, characterization and expression patterns
Source: BMC Plant Biol. 2022 Jan 3;22:2. doi: 10.1186/s12870-021-03374-y (PMC8722155; doi:10.1186/s12870-021-03374-y)

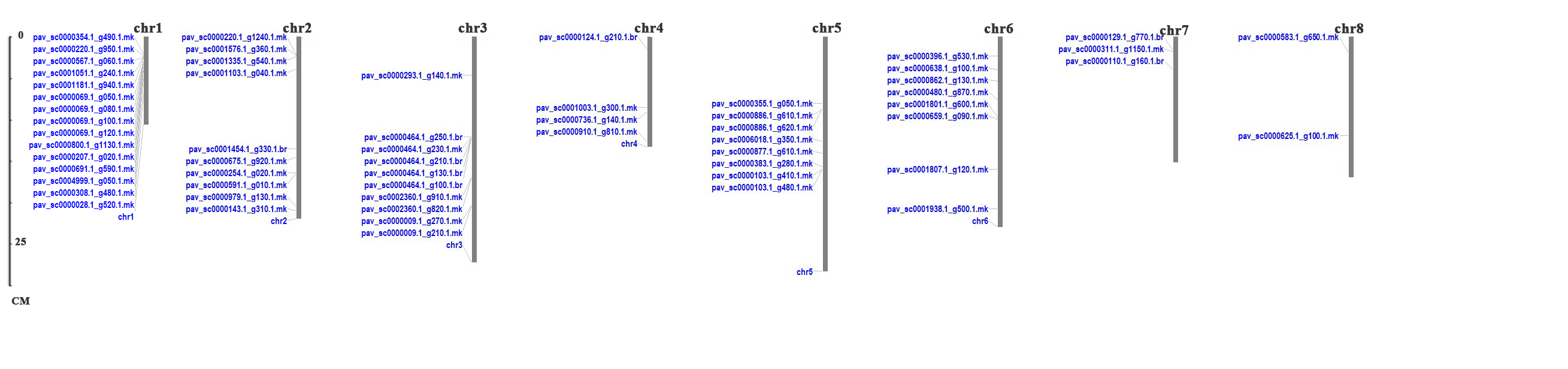

Supplement: Supplementary file 1 — Additional file 1: Figure S1. Nomenclature and chromosomal locations of MYB in sweet cherry. [file 12870_2021_3374_MOESM1_ESM.tif]

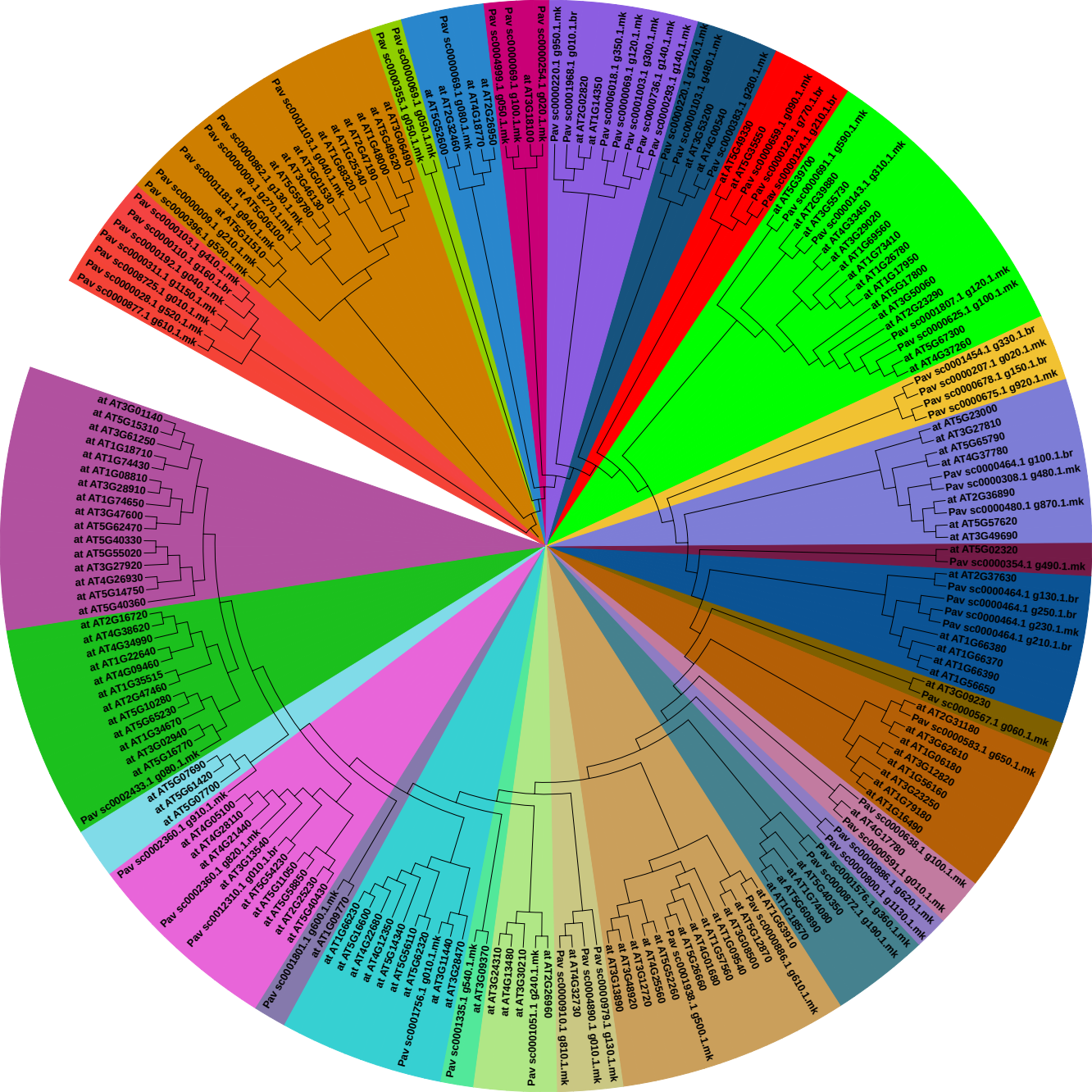

Supplement: Supplementary file 2 — Additional file 2: Figure S2. Phylogenetic tree of MYB subfamily from Prunus avium and Arabidopsis. Subgroups (C1-C28) of MYB subfamilies are highlighted with different colors. [file 12870_2021_3374_MOESM2_ESM.pdf]

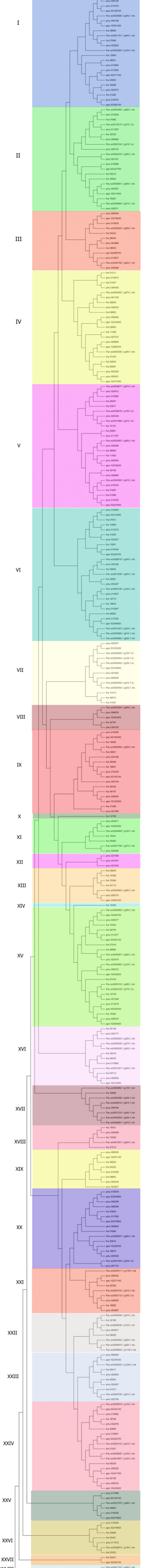

Supplement: Supplementary file 3 — Additional file 3: Figure S3. Phylogenetic tree of MYB subfamily from P. avium, F. vesca, P. mume, and P. persica. Subgroups (I-XXVIII) of MYB subfamilies are highlighted with different colors. [file 12870_2021_3374_MOESM3_ESM.pdf]
